# Brain and Behavioral Asymmetry: A Lesson From Fish

**DOI:** 10.3389/fnana.2020.00011

**Published:** 2020-03-26

**Authors:** Maria Elena Miletto Petrazzini, Valeria Anna Sovrano, Giorgio Vallortigara, Andrea Messina

**Affiliations:** ^1^School of Biological and Chemical Sciences, Queen Mary University of London, London, United Kingdom; ^2^Center for Mind/Brain Sciences, University of Trento, Rovereto, Italy; ^3^Department of Psychology and Cognitive Science, University of Trento, Rovereto, Italy

**Keywords:** behavioral lateralization, brain asymmetry, genetics, fish, zebrafish, drivers of lateralization

## Abstract

It is widely acknowledged that the left and right hemispheres of human brains display both anatomical and functional asymmetries. For more than a century, brain and behavioral lateralization have been considered a uniquely human feature linked to language and handedness. However, over the past decades this idea has been challenged by an increasing number of studies describing structural asymmetries and lateralized behaviors in non-human species extending from primates to fish. Evidence suggesting that a similar pattern of brain lateralization occurs in all vertebrates, humans included, has allowed the emergence of different model systems to investigate the development of brain asymmetries and their impact on behavior. Among animal models, fish have contributed much to the research on lateralization as several fish species exhibit lateralized behaviors. For instance, behavioral studies have shown that the advantages of having an asymmetric brain, such as the ability of simultaneously processing different information and perform parallel tasks compensate the potential costs associated with poor integration of information between the two hemispheres thus helping to better understand the possible evolutionary significance of lateralization. However, these studies inferred how the two sides of the brains are differentially specialized by measuring the differences in the behavioral responses but did not allow to directly investigate the relation between anatomical and functional asymmetries. With respect to this issue, in recent years zebrafish has become a powerful model to address lateralization at different level of complexity, from genes to neural circuitry and behavior. The possibility of combining genetic manipulation of brain asymmetries with cutting-edge *in vivo* imaging technique and behavioral tests makes the zebrafish a valuable model to investigate the phylogeny and ontogeny of brain lateralization and its relevance for normal brain function and behavior.

## Introduction

Brain lateralization is defined as the different functional specialization of the left and right sides of the brain. It was first described in the 19th century by M. Dax and P. Broca who showed that lesions in specific areas in the left hemisphere but not in the right one, were associated with deficits in producing language thus suggesting left hemisphere dominance for speech. For more than a century brain lateralization has been considered a human peculiarity linked to handedness, and complex cognitive functions, such as language (McManus, 1999; MacNeilage et al., 2009).

This belief has been challenged in the 1970s by a series of independent discoveries. Severing the left hypoglossal nerve impaired singing in songbirds, whereas severing the right nerve did not (Nottebohm, 1971). Unilateral hemispheric lesions in rats differently affected their exploratory behavior (Denenberg et al., 1978). Pharmacological treatment in the left hemisphere in chicks disrupted their visual discrimination abilities (Rogers and Anson, 1979). Since these first discoveries, the study of brain lateralization in non-human animals has become a burgeoning field of research and evidence of functional lateralization has been reported in species from all vertebrate classes (reviewed in see Vallortigara et al., 2011; Frasnelli et al., 2012; Ströckens et al., 2013; Ocklenburg et al., 2013; Rogers and Vallortigara, 2015; Vallortigara and Versace, 2017), thus demonstrating that it is a general feature of the animal brains (Rogers and Vallortigara, 2017). In particular, research on vertebrates has described a general pattern of lateralization among species, with the right hemisphere specialized in controlling social behavior, responding to novel and unexpected stimuli (e.g., predators) and processing global information whereas the left hemisphere is specialized to categorize stimuli, regulate routine behavior in familiar circumstances and focus attention to targets (Rogers et al., 2013; Rogers, 2014). For instance, a right-eye bias (left hemisphere dominance) for prey catching has been described in chicks (Mench and Andrew, 1986) pigeons (Güntürkün and Kesch, 1987; Güntürkün et al., 2000) and toads (Vallortigara et al., 1998; Robins and Rogers, 2004) and a left-eye bias (right dominance) in escape response to predators has been observed in dunnarts (Lippolis et al., 2005), horses (Austin and Rogers, 2007), lizards (Bonati et al., 2010, 2013), and toads (Lippolis et al., 2002).

Although investigation of brain lateralization in fish started more recently, data collected over the past 20 years have contributed much to the field (Vallortigara and Bisazza, 2002; Bisazza and Brown, 2011; Duboc et al., 2015). An advantage of using fish is due to the fact the eyes are laterally placed and the optic nerves decussate at the optic chiasm so that visual stimuli perceived with the right eye are predominantly processed by the left side of the brain and vice versa. As a consequence, it is possible to measure lateralized behavior in response to unilaterally presented stimuli and draw inferences about the functional specializations of the two hemispheres. Therefore, the observation of behavior represents a powerful non-invasive tool to assess the degree and direction of their brain lateralization.

Here we will provide a general overview of brain lateralization in fish. In particular, we will first present some examples of lateralized behaviors observed in the wild and in the laboratory highlighting the importance of these studies to comprehend the environmental impact on the development of asymmetrical biases and to understand the advantages and disadvantages of lateralized brains. We will then focus on the genetic mechanisms involved in the development of brain asymmetries discussing the relevance of zebrafish (*Danio rerio*) as a powerful animal model to link genetic, functional and behavioral asymmetries.

### Behavioral and Perceptual Asymmetries

There is considerable evidence of asymmetries in motor responses and sensory perception in fish (reviewed in Stancher et al., 2018; Table 1). An example of motor lateralization (i.e., behavioral bias at one of the two sides of the body) is represented by the fast escape response following threatening stimuli, more commonly known as C-start response. This response consists of a unilateral muscle contraction, which causes C-shape body bending, followed by a flip of the tail that allows the fish to flee from danger. This motor bias is triggered by the Mauthner cells, a pair of giant reticulospinal neurons that elicit muscle contraction and suppresses simultaneous activity of the opposite neuron thus allowing short response latencies (i.e., less than 20 ms; Domenici and Blake, 1997; Eaton et al., 2001; Korn and Faber, 2005). It has been showed that the right and left Mauthner neurons differ in size in the goldfish (*Carassius auratus*): individuals with the right larger neuron preferentially turn to the left side and vice versa thus suggesting that neuroanatomical asymmetry regulates the asymmetry of the C-start response (Moulton and Barron, 1967; Mikhailova et al., 2005). It has been suggested that asymmetry arises as a trade-off between direction and speed of escape responses (Vallortigara, 2000). Escape performance is fundamental for individual survival and strongly lateralized shiner perch (*Cymatogaster aggregata*) showed higher escape reactivity and superior ability to escape from predator attacks compared to non-lateralized fish (Dadda et al., 2010b). However, considerable variation in the direction of the fast start response has been observed. For instance, Heuts (1999) showed a population right-bias in C-start direction in zebrafish and goldfish while Lippolis et al. (2009) described a leftward population bias in a non-teleost fish, the Australian lungfish (*Neoceratodus forsteri*). By contrast, Bisazza et al. (1997a) and Izvekov and Nepomnyashchikh (2010) observed a bimodal distribution in the killifish (*Jenynsia multidentate*) and in the roach (*Rutilus rutilus)* with a similar number of individuals escaping to the left or to the right. Furthermore, a reversal in turning bias, from right to left, was observed in both juvenile and adult goldbelly topminnows (*Girardinus falcatus*) upon repeated presentations of a potential predator (Cantalupo et al., 1995) suggesting that the familiarity with the situation could lead the fish to perceive the stimulus as innocuous (since it never attacked the subjects) with shift toward control by the left side of the brain.

**TABLE 1:** Types of behavioral lateralization investigated and species in which lateralization has been observed or not.

Types of behavioral lateralization	Species	Occurrence of behavioral lateralization	References
**Motor asymmetry**			
Fast escape response	Goldfish (*Carassius auratus*)	Yes	Moulton and Barron, 1967; Mikhailova et al., 2005
	Shiner perch (*Cymatogaster aggregata*)	Yes	Dadda et al., 2010b
	Zebrafish *(Danio rerio)*	Yes	Heuts, 1999
	Australian lungfish (*Neoceratodus forsteri)*	Yes	Lippolis et al., 2009
	Killifish *(Jenynsia multidentata)*	Yes	Bisazza et al., 1997a
	Roach (*Rutilus rutilus*)	Yes	Izvekov and Nepomnyashchikh, 2010
	Goldbelly topminnows *(Girardinus falcatus)*	Yes	Cantalupo et al., 1995
	Giant danio (*Devario aequipinnatus*)	Yes	Stennett and Strauss, 2010
	Scissortail rasbora *(Rasbora trilineata)*	No	Stennett and Strauss, 2010
	Zebrafish *(Danio rerio)*	Yes	Stennett and Strauss, 2010
	White Cloud Mountain minnow (*Tanichthys albonubes*)	Yes	Stennett and Strauss, 2010
	Fathead minnow (*Pimephales promelas*)	Yes	Stennett and Strauss, 2010
Rotational swimming	Roach (*Rutilus rutilus*)	Yes	Izvekov and Nepomnyashchikh, 2010
	Mosquitofish *(Gambusia hoolbrooki)*	Yes	Bisazza and Vallortigara, 1996
	Sterlet sturgeon (*Acipenser ruthenus*)	Yes	Izvekov et al., 2014
	Roach (*Rutilus rutilus*)	Yes	Izvekov et al., 2012
Coiled posture	North eastern Pacific hagfish (*Eptatretus stoutii*)	Yes	Miyashita and Palmer, 2014
**Perceptual asymmetry**			
Foraging behavior	Zebrafish *(Danio rerio)*	Yes	Miklosi and Andrew, 1999; Hata and Hori, 2011
	Australian lungfish (*Neoceratodus forsteri*)	Yes	Lippolis et al., 2009
	Scale-eating cichlids (*Perissodus microlepis*)	Yes	Hori, 1993; Hori et al., 2007; Stewart and Albertson, 2010; Van Dooren et al., 2010; Lee et al., 2012; Takeuchi et al., 2012
	Cichlid *(Neolamprologus moori)*	Yes	Hori et al., 2007
	Freshwater goby (*Rhinogobius flumineus*)	Yes	Seki et al., 2000
	Japanese medaka (*Oryzias latipes*)	Yes	Hata et al., 2012
	Tanganyikan cichlid *(Julidochromis ornatus)*	Yes	Hata et al., 2012
	Scale-eating characiform (*Exodon paradoxu*s)	Yes	Hata et al., 2011
Social behavior	Mosquitofish (*Gambusia hoolbrooki*)		
	Females	Yes	Bisazza et al., 1998, 1999; Sovrano et al., 1999, 2001; De Santi et al., 2001
	Males	No	Bisazza et al., 1998; Sovrano et al., 1999
	Goldbelly topminnow (*Girardinus falcatus*)		
	Females	Yes	Bisazza et al., 1998
	Males	No	
	Convict cichlid (*Amatitlania nigrofas*)		
	Females	Yes	Moscicki et al., 2011
	Males	No	
	Breeding cichlid (*Neolamprologus pulcher*)	Yes	Reddon and Balshine, 2010
	Zebrafish *(Danio rerio)*	Yes	Sovrano et al., 2001; Sovrano and Andrew, 2006
	Redtail splitfin (*Xenotoca eiseni*)		
	Females	Yes	Sovrano et al., 1999
	Males	No	Sovrano et al., 1999
	Angelfish *(Pterophyllum scalare)*	Yes	Sovrano et al., 1999
	Eurasian minnow (*Phoxinus phoxinus*)	Yes	Sovrano et al., 1999
	Blue gourami (*Trichogaster trichopterus*)	Yes	Sovrano et al., 1999
	Sarasins minnow (*Xenopoecilus sarasinorum*)	Yes	Sovrano et al., 2001; Sovrano, 2004
	Elephantnose fish (*Gnathonemus petersii*)	Yes	Sovrano et al., 2001
	Soldierfish *(Myripristis pralinia)*	Yes	Roux et al., 2016
Mating behavior	Mosquitofish *(Gambusia hoolbrooki)*	Yes	Bisazza et al., 1998
	Goldbelly topminnow *(Girardinus falcatus)*	Yes	Bisazza et al., 1998
	Guppy *(Poecilia reticulata)*	Yes	Kaarthigeyan and Dharmaretnam, 2005
Agonistic behavior	Siamese fighting fish (*Betta splendens*)	Yes	Cantalupo et al., 1996; Bisazza and De Santi, 2003; Clotfelter and Kuperberg, 2007; Takeuchi et al., 2010; Forsatkar et al., 2015; HedayatiRad et al., 2017
	Mosquitofish *(Gambusia hoolbrooki)*	Yes	Bisazza and De Santi, 2003
	Redtail splitfin *(Xenotoca eiseni)*	Yes	Bisazza and De Santi, 2003

Note that in these studies it is difficult to discern the pure motor component from the behavioral bias that can be induced by visual lateralization. For instance, it is known that western mosquitofish (*Gambusia holbrooki*) and goldbelly topminnows preferentially use the right eye to monitor a predator when observed in the detour test, in which the fish had to swim along a runway until it faced a barrier behind which the predator was located, thus exhibiting a leftward turning bias (Bisazza et al., 1997b, 1998). However, the rightward turning preference described in four out of five species of minnows (Osteichthyes: Cyprinidae) observed in a T-shaped arena in the absence of any visual stimulus provided evidence of true motor asymmetries rather than behavioral lateralization induced by eye-use preference (Stennett and Strauss, 2010).

Rotational bias represents another example of motor asymmetry. When a fish is inserted in a circular environment it usually swims along its wall in either a clockwise or a counterclockwise direction even in the absence of any visual cue. Rotational biases have been found both in teleost species (Bisazza and Vallortigara, 1996; Izvekov and Nepomnyashchikh, 2010) and in chondrostean fish (Izvekov et al., 2014). Although the preferential direction of turning may be due to a specific eye preference to monitor the inner space (visual bias), this bias was also observed in the roach under infrared light (Izvekov et al., 2012) thus excluding visual lateralization as possible explanation of the asymmetrical activity. Despite studies on the Class Agnata (jawless fish) are very limited, motor lateralization has been described in the north eastern Pacific hagfish (*Eptatretus stoutii*). These eel-like, boneless, jawless, and sightless fish regularly rest in a tightly coiled posture but the clockwise or the counterclockwise coiling occurs equally often in the population (Miyashita and Palmer, 2014). The discovery of this behavioral bias in these fishes that are believed to be the most ancient group of living vertebrates (Ströckens et al., 2013) suggests that motor biases may represent the first evolutionary step for lateralization in vertebrates. Note, however, that studies on the lancelet, *Branchiostoma* (also known as amphioxus) provide key evidence for early asymmetry in chordate evolution. The mouth is on the left side of the body in larvae, but not in adults, meaning that the neural circuitry necessary to detect the prey are likely located on the left side of the brain. Despite the mouth is innervated by a nerve plexus that is on the left side of the larval brain, this connection is maintained also in the adults even if the mouth becomes frontal and symmetrical (Jeffries and Lewis, 1978). These data may explain the specialization of the left hemisphere to control feeding responses in vertebrates.

As mentioned, asymmetric behavioral responses can be attributed to lateralized processing of perceptual information (e.g., specific eye preferences to observe different classes of stimuli). For what concerns fish, research on brain lateralization has mainly focused on visual laterality rather than other sensory modalities (but see for an exception on fin use Bisazza et al., 2001a).

Behavioral preferences to attack a particular side of a prey and biases in foraging responses have been widely described in a variety of species. In the last decade, researchers showed an increased interest in studying the lateralization of foraging behavior from a behavioral, anatomical and genetic standpoint. For instance, zebrafish preferentially use the right eye when approach a target to bite (Miklosi and Andrew, 1999) and the Australian lungfish, which is considered to be the closest extant ancestor of tetrapods (Schultze, 1986), has been found to exhibit a rightwards bending of the body while feeding (Lippolis et al., 2009), in line with previous studies showing a left hemisphere dominance in controlling feeding behavior in several vertebrate species (see Andrew, 2002b for a review).

Among fish, scale-eating cichlids of genus *Perissodus* have become an attractive and useful model to study lateralization as they represent a striking example of interaction between morphological and behavioral asymmetries. These fishes exhibit jaw asymmetries that are dimorphic: individuals that open their mouth rightward preferentially attack the left side of their prey to tear off scales whereas fish that open the mouth leftward attack the right side (Hori, 1993; Takeuchi et al., 2012). This mouth-opening asymmetry has been described in other species (zebrafish, Hata and Hori, 2011; the cichlid *Neolamprologus moori*, Hori et al., 2007; the freshwater goby *Rhinogobius flumineus*, Seki et al., 2000; the Japanese medaka *Oryzias latipes* and the Tanganyikan cichlid *Julidochromis ornatus*, Hata et al., 2012; and a scale-eating characiform, *Exodon paradoxus*, Hata et al., 2011) and it has been advanced to be genetically determined by a one-locus two-allele Mendelian system, with the lefty dominant over the righty suggesting a common genetic basis in this morphological asymmetry among these species (Hori, 1993; Hori et al., 2007; Stewart and Albertson, 2010). However, a recent study on the scale-eating cichlid fish *Perissodus microlepis* has shown a strong behavioral bias even in laboratory-reared juveniles with relatively symmetrical mouth raising the hypothesis that mouth asymmetry is not a prerequisite for lateralized behavior but rather the preference to attack one side or the other may be expressed at an early age and may facilitate the development of the morphological asymmetry (Van Dooren et al., 2010; Lee et al., 2012). Future investigations are now required to better understand the relation between asymmetries in morphology and behavior, the mechanisms underlying the development of left-right axis and whether phenotypic plasticity contributes to shape the morphology in other species.

There is a large number of studies suggesting a right hemisphere dominance associated with social behavior in bird, mammals and amphibians. In fact, chicks show a left eye advantage in discriminating a familiar from an unfamiliar conspecific (Vallortigara and Andrew, 1991, 1994; Vallortigara, 1992) face recognition is mainly processed in the right hemisphere in primates (Hamilton and Vermeire, 1988) and sheeps (Kendrick, 2006; Versace et al., 2007) and five species of anuran amphibians preferentially use the left eye when looking at their own mirror image (Bisazza et al., 2002). Mirrors have been used to investigate visual lateralization in fish as well. Bisazza et al. (1999) studied cooperative predator inspection in female mosquitofish by inserting a mirror parallel to the tank at the end of which a predator was visible. In this way, the fish could see its own reflection when swimming along the mirror thus perceiving the presence of a cooperative partner. Mosquitofish were found to approach the predator more closely when the mirror was placed on the left side rather than on the right one, indicating a preferential use of the left eye when looking a conspecific. Consistent results were obtained using the mirror test, in which the mosquitofish were inserted in a tank with mirror walls, as the fish spent more time shoaling with the virtual companion when it was perceived on the left side (De Santi et al., 2001). The same left-eye preference has been observed in species belonging to different orders (Osteoglossiformes, Cypriniformes, Cyprinodontiformes, Beloniformes; Sovrano et al., 1999, 2001; Sovrano, 2004; Sovrano and Andrew, 2006) and also in females of a non-shoaling fish, the convict cichlid (*Amatitlania nigrofas*), but not in males. The authors suggested that despite the adults of this species do not form shoals, social experience early in development (during parental care) may have had lasting effects on lateralization in response to social stimuli (Moscicki et al., 2011). Interestingly, Reddon and Balshine (2010) described an opposite pattern in a highly social, cooperatively breeding cichlid fish (*Neolamprologus pulcher*) as males exhibited a right population preference to view their mirror image while females showed no significant population preference. It has been argued that the sex difference in eye use can be accounted to differences in social and sex motivation when viewing conspecifics. For instance, female mosquitofish and goldbelly topminnow exhibited a consistent rightward bias to detour a barrier to reach same sex conspecifics, whereas no preference was observed in males (Bisazza et al., 1998). Male mosquitofish did not show any eye preference during mirror-image inspection either (Sovrano et al., 1999). Note that females of both species are more social than males, which do not normally show social behavior and this may explain the absence of a side bias in response to social stimuli (Sovrano et al., 1999). Despite the absence of a behavioral bias in the detour task in presence of social companions (conspecifics of the same sex) in males topminnow and mosquitofish, a significant population bias has been observed when fish were presented with sexual stimuli (conspecific of the opposite sex) (Bisazza et al., 1998) whereas females showed a right-eye population biases for looking at the opposite-sex only when sexually deprived. Similarly, male-deprived female guppies (*Poecilia reticulata*) showed a stronger leftward turning bias in the detour (meaning right eye use) when viewing orange colored males than drab (Kaarthigeyan and Dharmaretnam, 2005).

Furthermore, lateralized perception of conspecifics may change as a function of familiarity. Juvenile soldierfish (*Myripristis pralinia*) with ablation of the left telencephalic hemisphere no longer displayed a preference toward conspecific versus heterospecifics fish but maintained this ability after the ablation in the right side of the brain thus showing that the left hemisphere was responsible for visual recognition of conspecifics (Roux et al., 2016). Right/left asymmetries to distinguish, respectively, familiar and unfamiliar conspecifics have been documented in different species (reviewed in Rosa Salva et al., 2012). Despite the direction of the lateralization in the soldierfish was reversed, the results provide further evidence of differential specialization of the two hemispheres in processing visual stimuli.

There is evidence that aggressive responses are mainly processed by the right hemisphere in many vertebrates (Rogers, 2002). For instance, gelada baboon (Casperd and Dunbar, 1996), chicks (Vallortigara et al., 2001), lizards (Hews and Worthington, 2001), and toads (Vallortigara et al., 1998) are more likely to attack a rival male on their left side than on their right side. In contrast to the previous examples, individual, but not population, lateralization in eye use during aggressive interactions (e.g., body posture) has been observed in male Siamese fighting fish (*Betta splendens*) when looking at their own reflection in a mirror (Cantalupo et al., 1996; Clotfelter and Kuperberg, 2007). Interestingly, the left or right preference was correlated with the morphological asymmetry in head incline; lefties (left-curved body) and righties (right-curved body) showed left- and right-biased eye use during aggressive displays, respectively (Takeuchi et al., 2010). However, Bisazza and De Santi (2003) described right population-level lateralization in mosquitofish, Siamese fighting fish and redtail splitfin (*Xenotoca eiseni*), suggesting that the difference observed with respect to the fighting fish might be due to different experimental conditions. The same right bias has been recently reported by Forsatkar et al. (2015) in nest-holding males fighting fish although the stages of reproduction and the paternal care affected the eye-preference with a shift from the right-eye to the left-eye after spawning. Similarly, exposure to an antidepressant drug (fluoxetine) reduced aggressive behavior and caused a change from a right to a left-eye use in this species even if the underlying mechanisms are still unknown (HedayatiRad et al., 2017).

All these data taken together indicate that eye preference when viewing conspecifics may stem from the natural history of the species but may also vary depending on the motivational state of the individuals that affects how certain information can be processed based on the context. It is clear that lateralization can be a highly flexible and complex phenomenon among species, within species and within individuals.

### Factors Involved in the Development of Brain Lateralization

It is widely acknowledged that genetic factors are involved in the establishment of lateralization. However, it is also clear that other environmental and physiological factors may play a crucial role in the development of brain asymmetry (reviewed in Deng and Rogers, 2002; Rogers, 2010, 2014). A well-known example is handedness in humans: although a genetic component has been reported for hand preference (Paracchini and Scerri, 2017), this behavioral bias can be modified as observed in different cultures where left-handers were pushed to “conform to normality,” that is right-handedness. This suggests that lateralization is a trait that results from the interplay between genes and environment (Cowell and Denenberg, 2002). There is compelling evidence in animals, that several environmental factors other than genetic mechanisms, modulate lateralization, such as light, hormones, rearing environment, pollution and stress (Table 2).

**TABLE 2:** Environmental factors that influence the development of lateralization.

Environmental factor	Species	Impact on lateralization	References
**Light stimulation**	Goldbelly topminnow (*Girardinus falcatus*)	Yes	Dadda and Bisazza, 2012
	Zebrafish (*Danio rerio)*	Yes	Budaev and Andrew, 2009; Sovrano et al., 2016
**Pollution** Elevated-CO2	Yellowtail demoiselle,	Yes	Domenici et al., 2012;
	(*Neopomacentrus azysron*)		Nilsson et al., 2012
	Clownfish *(Amphiprion percula)*	Yes	Nilsson et al., 2012
	Spiny damselfish (*Acanthochromis polyacanthus*)	Yes	Jarrold and Munday, 2018
	Three-spined stickleback (*Gasterosteus aculeatus*)	Yes	Jutfelt et al., 2013
	Sand smelt (*Atherina presbyter*)	Yes	Lopes et al., 2016
	Zebrafish *(Danio rerio)*	Yes	Vossen et al., 2016
	Two-spotted gobies (*Gobiusculus flavescen*s)	Yes	Sundin and Jutfelt, 2018
	Copper rockfish (*Sebastes caurinus*)	Yes	Hamilton et al., 2017
	Goldsinny wrasse (*Ctenolabrus rupestris*)	No	Sundin and Jutfelt, 2015
	Atlantic cod (*Gadus morhua*)	No	Jutfelt and Hedgärde, 2015
	Blue rockfish (*Sebastes mystinus*)	No	Hamilton et al., 2017
	Damselfish, (*Pomacentrus wardi)*	Yes	Domenici et al., 2014
Warming	Damselfish, (*Pomacentrus wardi)*	Yes	Domenici et al., 2014
Anthropogenic noise	European eels (*Anguilla anguilla*)	Yes	Simpson et al., 2015
Chemical pollutants	Surgeonfish *(Acanthurus triostegus)*	Yes	Besson et al., 2017
Hypoxia	Staghorn sculpin *(Leptocottus armatus)*	Yes	Lucon-Xiccato et al., 2014
**Rearing environment**	Rainbowfish *(Melanotaenia duboulayi)*	Yes	Bibost et al., 2013
	Guppy *(Poecilia reticulata)*	Yes	Broder and Angeloni, 2014; Dadda and Bisazza, 2016
	Poeciliid *(Brachraphis episcopi)*	Yes	Brown et al., 2004, 2007
	Whitetail damsels (*Pomacentrus chrysurus*)	Yes	Ferrari et al., 2015
	Yellow-and-blueback fusiliers (*Caesio teres*)	Yes	Chivers et al., 2016
**Stressor factors**	Yellowtail demoiselle, (*Neopomacentrus azysron*)	Yes	Domenici et al., 2012
	Three-spined stickleback (*Gasterosteus aculeatus*)	Yes	Jutfelt et al., 2013
	Small-spotted catsharks (*Scyliorhinus canicular*)	Yes	Green and Jutfelt, 2014
	Arctic charr (*Salvelinus alpinus*)	Yes	Backstroöm et al., 2015
	Poeciliid *(Brachraphis episcopi)*	Yes	Brown et al., 2007
	Goldbelly topminnow *(Girardinus falcatus)*	Yes	Dadda et al., 2007

#### Genetic Mechanisms

Clear evidence of heritability of lateralization was provided by Bisazza et al. (2000b) using artificial selection experiments in goldbelly topminnows. Males and females were initially tested on a detour task for their eye-preference to inspect a predator. Only males and females with similar high laterality scores were mated together and, then, their progeny was tested in the same test. Offspring exhibited the same behavioral biases observed in their parents (e.g., the progeny of the right-eye fish, used the right eye to monitor the predator) showing that lateralization was inherited both in strength (i.e., an individual can be more or less lateralized) and direction (left or fight). Furthermore, subsequent studies demonstrated that belonging to these lines selected on the basis of their eye preference to monitor a threating stimulus was predictive of behavioral lateralization in other tasks (e.g., eye used by males in sexual behavior or agonistic attacks), suggesting that these fish may have a mirror-reversed organization of cerebral specializations (Bisazza et al., 2001b, 2005; Dadda et al., 2007, 2009, 2012). We will then show how sophisticated molecular and genetic techniques have been used in zebrafish to address the role of genes in the establishment of brain asymmetry.

#### Hormones

Steroid hormones have been suggested to affect brain lateralization in humans and non-human animals (Beking et al., 2017). However, data collected in humans are ambiguous and the effect of hormones on the development of lateralization is still heavily disputed suggesting that animal models could be useful to allow experimental manipulation not feasible in humans. For instance, the injection of testosterone and corticosterone *in ovo* altered the development of the asymmetry of thalamofugal visual pathway in chicks (Schwarz and Rogers, 1992; Rogers and Deng, 2005). Reddon and Hurd (2008) observed that convict cichlids males (*Archocentrus nigrofasciatus*) were more lateralized when looking at an aversive stimulus whereas females where more lateralized when looking at a stimulus associated to a positive reinforcement thus suggesting a potential effect of hormones on visually guided behavioral lateralization. Recently, Schaafsma and Groothuis (2011) directly investigated, in fish, the impact of postnatal testosterone on the eye preference when inspecting a predator. Results showed a right-eye population bias to monitor the predator only in fish treated with testosterone, but not in control fish. Furthermore, males were more responsive to the treatment providing first evidence of an involvement of hormones also in fish lateralization. However, the relation between lateralization and steroid hormones is still unclear and future studies may be of help to better understand the role of hormones in modulating brain and behavioral asymmetries.

#### Light Stimulation

Exposure to light represents one of the best described examples of environmental factors affecting brain lateralization. Light stimulation plays a crucial role in the asymmetrical development of the visual pathway in the domestic chick. Infact, chick position within the eggs determines which eye receives light stimulation through the shell (Rogers, 1990, 1997). Chicks with either the left or the right eye covered develop a reversed pattern of asymmetry whereas chicks from eggs incubated in the dark do not exhibit any asymmetry (Rogers, 2008). This neuroanatomical asymmetry is reflected on the behavior as chicks hatched from eggs with their left eye occluded used the right eye (left hemisphere) to distinguish food from pebbles and the left eye (right hemisphere) to monitor a predator; the behavioral asymmetry is reversed in chick hatched from eggs with the right eye occluded (Rogers, 2008, 2014; Chiandetti and Vallortigara, 2009, 2019; Vallortigara et al., 2011; Chiandetti et al., 2013, 2017). The amount of environmental light received during the development influences lateralization in fish too. Pre-natal effect of light exposure has been observed in live-bearing fish by exposing females goldbelly topminnow to either high or low intensity of light during pregnancy. Only progeny from the light group developed behavioral asymmetries in a visual and motor task (Dadda and Bisazza, 2012). Budaev and Andrew (2009) found that light vs. dark incubation of zebrafish embryos determined eye preference for avoiding a predator. Embryos exposed to light kept at greater distance when a potential predator was seen with the left rather than right eye whereas this behavioral asymmetry was reduced in dark-incubated zebrafish. However, light exposure had a differential effect during the first few days of development as absence of light on day 1 reversed eye-preference but the shift was reduced in absence of light on day 2 or 3 indicating a sensitive period for the effect of light. Although the authors suggested that early light stimulation may affect expression of genes involved in the asymmetric development of the habenulae, subsequent studies showed that darkness delays neurogenesis in the habenular nuclei but does not eliminate asymmetric gene expression (De Borsetti et al., 2011). Recently, Sovrano et al. (2016) showed that only zebrafish exposed to natural light/dark (LD) cycle, developed a left-eye preference in the mirror test but not zebrafish exposed to different wavelengths of light suggesting an effect of lighting condition on development of social recognition.

#### Pollution

In the last decade, a new environmental factor has been added to the list of agents modulating the development and expression of lateralization: pollution.

Ocean acidification is caused by increased concentration of CO_2_ dissolved into the water due to the rise in anthropogenic-related atmospheric CO_2_. Growing evidence now indicates that elevated-CO_2_ concentrations can alter behavior and sensory abilities of larval and juvenile fishes (e.g., fish are attracted by chemical that they usually avoid) and also affect lateralized behavior (Munday et al., 2009; Simpson et al., 2011; Cattano et al., 2018; Esbaugh, 2018). Several studies have shown that exposure to elevated-CO2 causes loss of lateralization both in coral reef fish (yellowtail demoiselle, *Neopomacentrus azysron*; clownfish, *Amphiprion percula*; spiny damselfish, *Acanthochromis polyacanthus*) and in temperate fish (three-spined stickleback, *Gasterosteus aculeatus*; sand smelt, *Atherina presbyter*, zebrafish; two-spotted gobies, *Gobiusculus flavescen*s) potentially having negative consequences for survival in natural habitats by increasing vulnerability to predators and affecting social cohesion (Domenici et al., 2012; Nilsson et al., 2012; Jutfelt et al., 2013; Lopes et al., 2016; Vossen et al., 2016; Jarrold and Munday, 2018; Sundin and Jutfelt, 2018). Loss of behavioral lateralization induced by elevated CO2 is restored by treatment with an antagonist of the GABA-A receptor, suggesting that high level of CO2 interferes with neurotransmitter function (Nilsson et al., 2012; Lai et al., 2015; Lopes et al., 2016). Taken together, these studies indicate that ocean acidification could represent a problem that affects fish on a global scale.

However, there are species-specific differences in tolerance to increased level of CO_2_. No effect of CO_2_ on behavioral lateralization has been reported in goldsinny wrasse (*Ctenolabrus rupestris*) (Sundin and Jutfelt, 2015), juvenile Atlantic cod (*Gadus morhua*) (Jutfelt and Hedgärde, 2015) and in Blue rockfish (*Sebastes mystinus*) although changes in behavioral lateralization have been described in the phylogenetically closely related species the Copper rockfish (*S*. *caurinus*) (Hamilton et al., 2017). Differences in response to CO_2_ may be due to increased adaptive response in some species compared to others. Future studies are now required to understand whether and to what extent species have the capacity to adapt to elevated CO_2_ to make good predictions about the ecological consequences of ocean acidification.

Other stressors that disrupt lateralization are ocean warming (Domenici et al., 2014), anthropogenic noise in aquatic environments (e.g., commercial shipping and recreational boating) (Simpson et al., 2015), chemical pollutants added to water (i.e., pesticide) (Besson et al., 2017) and hypoxia that is exacerbated by human activities (e.g., agriculture and discharge of raw sewage) that increases coastal eutrophication (Lucon-Xiccato et al., 2014). It is clear from these studies that some environmental factors affecting lateralization are due anthropic activities and that management and policy decisions are needed to reduce their negative effects on fish behavior that can, in turn have severe implications for community structure and ecosystem function.

#### Rearing Environment

Early visual experience has been found to influence behavioral lateralization. Bibost et al. (2013) investigated the role of environmental complexity by rearing rainbowfish (*Melanotaenia duboulayi*) in enriched and impoverished conditions and found that males from impoverished habitats were more lateralized than males from enriched environment in their schooling behavior. Females, instead, showed the opposite pattern. Recently, Dadda and Bisazza (2016) showed that newborn guppies raised in an asymmetric environment exhibited eye preference in the mirror test congruent with the direction of asymmetric stimulation suggesting that early different exposure to left/right visual information affects the development of brain asymmetries. Several studies have documented that predation pressure represents another key factor determining the degree of lateralization. The poeciliid *Brachraphis episcopi* reared in high predation environments showed a different pattern of visual lateralization compared to fish from low predation areas (e.g., right eye use to monitor a predator compared to non-visual lateralization; Brown et al., 2004, 2007) and guppies exposed to olfactory predator cues were more highly lateralized than conspecifics reared in absence of threatening cues (Broder and Angeloni, 2014). In line with previous findings, juvenile whitetail damsels (*Pomacentrus chrysurus*) exposed to alarm cues (i.e., injured conspecific cues that elicited an antipredator response) displayed increased behavioral lateralization compared to low-risk condition fish (Ferrari et al., 2015). Chivers et al. (2016) also found that predation pressure affected the strength of lateralization in juvenile yellow-and-blueback fusiliers (*Caesio teres*) and hypothesized that predation stress induced phenotypic plasticity of lateralization which is likely to be adaptive as higher degree of asymmetries increases fitness and survival in environments with high predation risk.

Despite the field of research on environmental mechanisms affecting lateralization is expanding, only the interaction between genetic and environmental factors may provide a clear picture of the relative contribution of these drivers in determining brain asymmetries.

#### Stress

Stressor factors could influence brain lateralization in fishes (Brown et al., 2007; Dadda et al., 2007; Jutfelt et al., 2013; Domenici et al., 2014; Green and Jutfelt, 2014; Backstroöm et al., 2015) as in other animal species (Ocklenburg et al., 2016). Data from literature showed that increased levels of carbon dioxide were associated to decreased behavioral lateralization in the yellowtail demoiselle (Domenici et al., 2012) and in the three-spined stickleback (Jutfelt et al., 2013) whereas an opposite effect was reported in small-spotted catsharks (*Scyliorhinus canicular;* Green and Jutfelt, 2014). Among the other stressor factors, social interaction (Arctic charr, *Salvelinus alpinus*: Backstroöm et al., 2015), predation pressure (*Brachraphis episcopi*: Brown et al., 2007) and the introduction in a new environment (goldbelly topminnows: Dadda et al., 2007) could also affect behavioral lateralization in different fish species. Beside individual variations in response to stressful environments, the relationship between stress-reactivity and laterality has also been investigated. For instance, it has been shown that the degree of laterality in Port Jackson sharks (*Heterodontus portusjacksoni*) was correlated with stress and stronger lateralized individuals were more reactive (Byrnes et al., 2016), whereas this correlation has not been observed in zebrafish (Fontana et al., 2019). However, research on the relation between laterality and stress is still in the early stages and further investigation is essential to understand the role of steroid hormones (i.e., glucocorticoids) in modulating functional hemispheric asymmetries.

### Pros and Cons of Asymmetric Brains

The ubiquitous nature of lateralization suggests that it confers advantages on the individuals. For instance, the specialization of each hemisphere in controlling different functions is supposed to prevent the simultaneous activation of incompatible responses leading to more efficient information processing and rapid responses (Andrew, 1991; Vallortigara, 2000). Furthermore, it allows sparing neural tissue by avoiding duplication of functions in the two hemispheres and increases neural capacity (Levy, 1977; Vallortigara and Rogers, 2005). Another benefit related to lateralization consists of the capacity to simultaneously process multiple types of stimuli. As a consequence, lateralized individuals may carry out different tasks in parallel thus coping better with situations involving divided attention (Rogers, 2000; Vallortigara and Rogers, 2005). This hypothesis was first tested in chicks (Rogers, 2014) and then has been confirmed in fish by comparing lateralized and non-lateralized topminnows while performing two simultaneous tasks: predator vigilance and prey capture. Lateralized individuals were faster at capturing prey in the presence of a predator as they monitored it with one eye and used the opposite eye for catching prey, whereas non-lateralized fish continuously switched from one eye to the other for both functions (Dadda and Bisazza, 2006a). Similarly, lateralized female topminnows foraged more efficiently than non-lateralized females when they had to attend to a harassing male at the same time (Dadda and Bisazza, 2006b).

Evidence for the hypothesis that lateralization is linked to enhanced performances comes from studies showing higher cohesion and coordination in schools of lateralized topminnows than in schools of non-lateralized fishes. Furthermore, lateralized individuals were located in the center of the school, a position normally safer and energetically less expensive, whereas non-lateralized fish were at the periphery (Bisazza and Dadda, 2005). Recently, Bibost and Brown (2013) observed in two rainbowfish species (*Melanotaenia duboulayi* and *Melanotaenia nigrans*) that individuals occupied positions in the schools based on the eye they preferentially used to monitor conspecifics: fish with a left-eye bias adopted a position on the right side of the school, whereas fish with a right-eye bias were located on the left side of the school. These data confirm that lateralization influences schooling behavior allowing individuals to process visual information more quickly in the appropriate hemisphere. Furthermore, lateralized topminnows have been found to reorient themselves better than non-lateralized conspecifics using both geometric and non-geometric cues thus showing improved spatial navigation skills (Sovrano et al., 2005).

Cerebral lateralization conveys a selective advantage also by increasing learning abilities. Rainbowfish selected on the basis of the eye used to monitor their mirror image, where trained to associate a red light with a food reward using a classical conditioning paradigm. Despite the authors did not include non-lateralized individuals, they found that left-lateralized fish learned the task faster compared to right-lateralized in line with the idea that cognitive abilities may be influenced by the degree of laterality (Bibost and Brown, 2014). Dadda et al. (2015) found that strongly lateralized guppies selected in the mirror test, out-performed non-lateralized guppies in two numerical tasks: both when they were trained to discriminate between arrays containing a different number of geometric figures and when they were observed in a spontaneous choice task for their preference to join the larger of two shoals. No difference between fish with left or right-eye use was found. Consistent results were obtained in the shoal choice task when guppies where selected for high or low lateralization using the detour test in presence of a predator rather than the mirror test (Gatto et al., 2019). Numerical abilities are linked to brain lateralization also in the threespine stickleback as fish tested in a shoal choice task in monocular condition (i.e., with one eye covered) performed better than fish observed in binocular condition (i.e., no eyes covered) but only when presented with certain numerical contrasts. One possible explanation of the better performance in the monocular fish may be the absence of conflicting responses from the two hemispheres that allowed more effective information processing when making a choice (Mehlis-Rick et al., 2018).

Despite clear advantages, lateralization can also provide costs to the fitness of organisms. In natural environments the position of predators, prey and competitors is unpredictable as they can appear on both sides of an individual. Hence, lateralized organisms can be more vulnerable to attacks or miss feeding opportunity if the stimuli are not perceived in the “preferred” visual hemifield (Rogers et al., 2004). The hypothesis that lateralization can hinder performance has been tested in tasks that requires interhemispheric communication by comparing lateralized and non-lateralized topminnows in two tests: a bisection-like test and a shoal choice test (Dadda et al., 2009). In the former experiment, fish were trained to select the middle door in a row of nine in order to join their social companions. Non-lateralized individuals performed better as they chose more often the central door, whereas lateralized individuals systematically chose the door on the left or right of the correct one. In the second experiment, the fish were presented with two shoals of conspecific differing in quality (number and size of fishes) placed in a way that each of them was visible in a different visual hemifield. Non-lateralized fish chose the high-quality shoal but lateralized fish selected the shoal on the side of the eye dominant for analyzing social stimuli. In both cases, the suboptimal choices observed have been attributed to the lack of integration of information between the hemispheres, as if visual information remained confined to the hemisphere that initially received it.

The advantages provided by lateralization explain why asymmetries can occur at “individual-level” (e.g., each individual exhibits its own directional bias but left and right asymmetries are equally distributed within the population) but not why lateralization often occurs at a population-level (i.e., the majority of individuals within the population exhibits the same directional bias) (Vallortigara, 2006a). A problem arises as lateralization at the population level can make individual behavior more predictable: a predator can exploit the fact that prey show a preferential escape direction and can attack on the other unexpected side to increase its success. Similarly, predators specialized in a lateralized attack might increase their capture success but prey can learn how to avoid them. In this scenario, it would be better to have a 50:50 distribution of right and left lateralized individuals within a population. For instance, group hunting sailfish show individual-level lateralization when attacking shoals of sardines but the collective predictability is minimized by random group assortment and attack alternation so that each individual only sporadically performs multiple attacks (Herbert-Read et al., 2016). It is possible then, that group hunting may represent a condition that favors the evolution of individual-level lateralization (Kurvers et al., 2017). Foraging behavior in scale-eating cichlid fish represents another example in which selective pressures favored equal distribution of behavioral bias as the proportion of left and right lateralized individuals oscillates around a 50:50 ratio (Lee et al., 2012).

Ghirlanda and Vallortigara (2004) developed a theoretical model showing that in the context of pray-predator interactions and competitive-cooperative interactions, population-level lateralization may have arisen as an evolutionary stable strategy (ESS) when it is more advantageous for individually lateralized organisms to align their biases to the direction of other asymmetrical organisms to coordinate their behavior with them (Vallortigara and Rogers, 2005; Ghirlanda et al., 2009). According to this model, the alignment of the direction of lateralized biases in a population may have evolved when the individuals experienced greater benefits when performing the same behavioral tactic (Vallortigara, 2006b). Hence, “social” species would be expected to be lateralized at the population level whereas “solitary” species at the individual level only. Empirical evidence in support of this hypothesis comes from a study by Bisazza et al. (2000a) showing that behavioral biases at population level were more frequent in social fish species than in solitary ones.

It is clear that escaping in the same direction reduces the chance of each individual within the groups to be caught by the predator because of the “dilution effect” (i.e., it is more difficult for the predator to target a specific individual). However, as mentioned before, predators can learn how to anticipate the behavior of the pray. As a consequence, it would be better for some individuals to escape in the opposite unexpected direction to increase their chance of survival. Therefore, a combination of opposite selective forces (i.e., the need for coordination and the need for unpredictability) seems to play a crucial role in guiding the alignment of the direction of asymmetries. The successful strategy for group-living pray would consist in joining the majority to gain protection with a minority that increases its chance to survive by surprising the predator. But how can we explain the existence of majority and minority biases with respect to laterality? It has been suggested that lateralization at population level may be under effect of frequency-dependent selection, a process in which the advantage of one phenotype (e.g., right biased individuals) depends on its frequency in relation to the other phenotype (e.g., left biased individuals) and the advantage would disappear when the minority increases in number (Connor and Hartl, 2004). Frequency-dependent selection does in fact emerge spontaneously as an ESS in Ghirlanda and Vallortigara (2004)’s model. Loffing (2017) showed that left-handers are more successful in competitive sports that reflect some elements of fighting (e.g., boxing, fencing) and proposed that left-handedness in humans is maintained by frequency-dependent mechanisms. In case of fish, despite two-thirds of mosquitofish preferentially use the right eye to monitor the predator, the remaining third used the left eye (De Santi et al., 2001), confirming advantages associated with the existence of the minority type of lateralization.

In conclusion, it is possible to argue that the advantages associated with lateralization counteract the possible disadvantages and that the trade-off between costs and benefit would account for the presence of a certain proportion of non-lateralized individuals in animal populations and for the maintenance of population-level lateral biases (Bisazza et al., 1997a, 2000a; Güntürkün et al., 2000; Brown et al., 2007; Takeuchi and Hori, 2008; Frasnelli and Vallortigara, 2018; Vallortigara, 2019; Vallortigara and Rogers, 2020).

### Brain Asymmetry in Zebrafish: Insight From Habenular Nuclei

In the last 20 years, zebrafish has become an excellent model to study the central nervous system (CNS) lateralization due to many advantages it offers in term of body transparency, small size, rapid embryonic development and genetic manipulation (Kalueff et al., 2014; Stewart et al., 2014). As a consequence, zebrafish represents a powerful tool to look inside the developmental and functional basis of brain asymmetry following a comprehensive bottom-up approach (from genes to behavior) and vice versa (from behavior to genes) (Duboc et al., 2015).

The most pronounced structural asymmetry in zebrafish brain was found in the epithalamus. The epithalamus is the dorsal part of the vertebrate diencephalon and displays a marked structural and functional left-right asymmetry that is conserved in a large number of vertebrates (Concha and Wilson, 2001; Bianco and Wilson, 2009; Aizawa et al., 2011). For example, fish and mammalian habenulae are considered to be homologous structures as they are subdivided into a lateral and a medial domain in both taxa (Amo et al., 2010), but they are anticlockwise rotated by 90° compared to each other (Güntürkün and Ocklenburg, 2017). In detail, zebrafish epithalamus contains an unpaired pineal complex, medially positioned, and two bilateral habenular nuclei (Bianco and Wilson, 2009; Aizawa et al., 2011; Güntürkün and Ocklenburg, 2017). It has been established that there is asymmetric termination of forebrain neurons in the habenulae and that there are left/right asymmetries in the efferent connectivity of the habenular nuclei with the interpeduncular nucleus (IPN) in the midbrain, suggesting a conserved connecting system between forebrain and ventral midbrain across vertebrates (Bianco and Wilson, 2009; Miyasaka et al., 2009; Aizawa et al., 2011; Beretta et al., 2012; Roussigné et al., 2012). Furthermore, connectional asymmetries in zebrafish epithalamus are recognizable at the level of pineal complex. The pineal complex is composed by two main structures: a pineal and a parapineal organ (Concha and Wilson, 2001). The pineal is a photosensitive gland, medially positioned, involved in the release of melatonin and in the circadian clock and it does not generate any symmetric/asymmetric connection with the lateral habenular complex. On the contrary, the parapineal complex is the second example of asymmetry in zebrafish epithalamus since it is located on the left side respect to the pineal gland and projects only to the lateral subnucleus of the left dorsal habenula (Concha et al., 2000; Gamse et al., 2005).

### Molecular Mechanisms Regulating the Development of Epithalamic Asymmetry in Zebrafish

**FIGURE 1:**
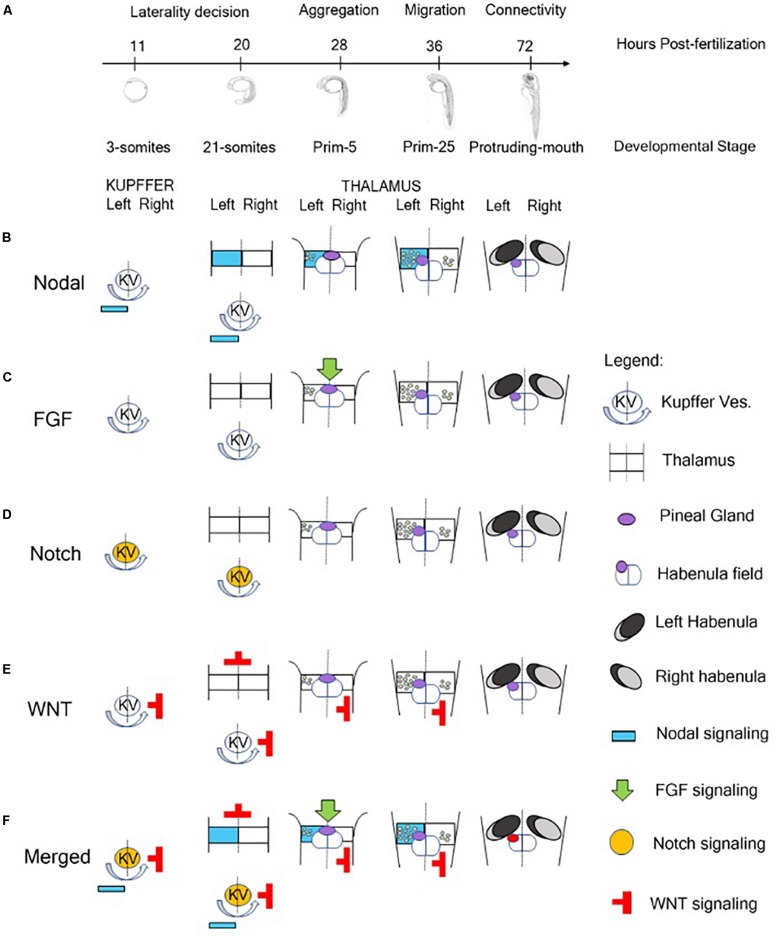
Role of signaling pathway in the Generation of Neuroanatomical Asymmetry in zebrafish. **(A)** Timeline of developmental stages involved in the epithalamic lateralization in zebrafish. **(B)** Nodal signaling influences left-right asymmetry starting from 3-somites stage in which Kupffer’s Vesicle contributes to the positioning of Nodal-related genes on the left side of zebrafish embryo (Raya et al., 2003). At 28 hpf, with the aggregation of the symmetric parapineal cells on the midline of epithalamus, the forming pineal complex becomes asymmetric with the migration of parapineal cells in the left side of the brain where Nodal-related genes contribute to the differentiation of left-sided habenular nuclei (Concha et al., 2000; Long et al., 2003; Carl et al., 2007; Inbal et al., 2007; Snelson and Gamse, 2009; Roussigné et al., 2012; Duboc et al., 2015). During later development, Nodal signaling is also involved in the generation of connectivity of epithalamic structures (Hüsken et al., 2014). **(C)** At 28 hpf, FGF signaling plays a role in breaking the symmetry of the brain contributing to the positioning of Nodal-related genes on the left side of the embryo (Neugebauer and Yost, 2014). **(D)** Notch pathway is involved in the control of cilia length of Kupffer’s Vesicle responsible for breaking the initial symmetry generating a directional fluid flow from the Kuppfer’s Vesicle to the left side of the zebrafish embryo and to positioning Nodal signaling molecules on the left side (Hashimoto et al., 2004; Gourronc et al., 2007; Hojo et al., 2007). **(E)** The Wnt/beta-catenin cascade acts in the lateral mesodermal plate before the induction of Nodal pathway components contributing to the establishment of left-right asymmetry of the brain in three different developmental stages of zebrafish: late gastrulation, somitogenesis and during epithalamic development (Carl et al., 2007; Hüsken and Carl, 2013). **(F)** In brief, Notch signaling influences the direction of fluid flow originated by ciliated cells of Kupffer’s vesicle and contributes to the positioning of Nodal-related genes on the left side of zebrafish embryo and of Nodal inhibitors and WNT signaling molecules on the right. At later stage, FGF signaling breaks the symmetry of the epithalamic structures and, in synergy with Nodal pathway, plays a role in the establishment of brain asymmetry in zebrafish embryo contributing to the migration of parapineal cells on the left side and to the generation of asymmetric habenular nuclei.

The molecular events that regulate the development of asymmetric structures in the dorsal forebrain and, in particular, in the epithalamus of vertebrates are still partially unknown. Data collected in zebrafish showed an involvement of four major pathways in the establishment of epithalamus asymmetry: Nodal, Fibroblast Growth Factors (FGFs), Notch and Wnt/beta catenin (Hüsken and Carl, 2013; Figure 1).

During embryonic development of zebrafish, the epithalamus evolves as a bilateral symmetric structure that is subdivided in a dorsal and ventral domain that become asymmetric when the component of Nodal signaling pathway arrived from the dorsal and lateral mesoderm (Concha et al., 2003; Figure 1B). Different studies reported that mutant zebrafish lines lacking of notochord express the nodal-related gene *cyclops* (*cyc*, also called *ndr2*) bilaterally in the dorsal diencephalon, suggesting an involvement of the dorsal mesoderm in the development and maintenance of zebrafish epithalamus asymmetry (Rebagliati et al., 1998; Sampath et al., 1998; Bisgrove et al., 2000). The nodal-related genes (*squint* and *cyclops*, in particular) start to be expressed during zebrafish gastrulation in the dorsal and lateral mesoderm driving the ventral neuroectoderm to acquire floorplate identity (Erter et al., 1998; Rebagliati et al., 1998; Sampath et al., 1998). Furthermore, Liang et al. (2000) established that these mesodermal signals could be required to position and preserve the left-sided gene expression in forebrain and, in particular, in the diencephalon. These authors localized the co-expression of *cyclops*, *antividin* (*atv*, a *lefty1*-related gene) and *pitx2* (a nodal-related transcription factor) in the left dorsal side of diencephalon in the region in which the medial invagination, forming the pineal complex, originates. Using an RNA-mediated rescue approach, they were also able to recover pineal complex structures in adult fish generated starting from mutant embryos lacking the left-sided expression of *cyc*, *atv*, or *pitx2*. Moreover, they reported that the pineal complex of these fishes was frequently displaced on the right of the epithalamus midline, proposing that the Nodal pathway was essential during zebrafish early embryogenesis to position the parapineal domain and resulting organs on the left side of zebrafish brain midline. These data suggested that signaling pathways regulating visceral laterality were also able to produce anatomical asymmetry of zebrafish forebrain (Liang et al., 2000).

Other important evidences of an involvement of Nodal signaling in the generation of asymmetric epithalamic structures derived from the earliest stages of habenular development in zebrafish. Roussigné et al. (2009) focused their attention on a habenular progenitor marker, named *cxcr4b* (*C-X-C chemokine receptors 4b*), which is expressed in the habenular progenitors prior to the leftward migration of parapineal cells. The removal of left/right bias, induced by Nodal signaling, was able to generate symmetric habenular nuclei promoting the idea of a role of this pathway as a guide for the development of brain asymmetry, rather than only for directing laterality. These data were also supported by evidence that SB431542, a chemical inhibitor of Nodal pathway, was able to alter epithalamus asymmetry in favor of the generation of symmetric or mild asymmetric structures compared to untreated controls. These results confirmed previous studies showing that the knock-down of Southpaw (another early mesodermal nodal-related gene) in zebrafish embryos resulted in a severe downregulation of left-sided expression of *cyclops*, *pitx2*, *lefty1*, and *lefty2* in the dorsal epithalamus (Long et al., 2003; Barth et al., 2005; Roussigné et al., 2009). Taken together, these data suggest that, in zebrafish, Nodal signaling derived from the dorsal and lateral mesoderm is responsible for the expression of nodal-related genes (*ndr2* or *cyclops*) on the left side of epithalamus orchestrating the leftward migration of parapineal cells and, consequently, the generation of the asymmetric structures in the brain through the transcription of feedback inhibitor *lefty1* and the homeodomain transcription factor *pitx2c* (Concha et al., 2000; Long et al., 2003; Carl et al., 2007; Inbal et al., 2007; Snelson and Gamse, 2009; Roussigné et al., 2012; Duboc et al., 2015).

Although the Nodal pathway plays a pivotal role in the generation of forebrain asymmetry, FGF signaling represents the initial driving force (Figure 1C). Regan et al. (2009) showed that the leftward migration of parapineal complex was driven by FGF8. In fact, zebrafish FGF8 mutant embryos, also called *acerebellar*, or FGF8 morphants are not able to develop epithalamic and habenular asymmetry, because parapineal cells fail to migrate resulting in a symmetric structure (Reifers et al., 1998; Draper et al., 2001; Regan et al., 2009). These data were also supported by experiments of chemical inhibition of FGF receptors. In fact, the temporally inhibition of FGF signaling through the drug SU5402, disrupted parapineal migration blocking parapineal cells closure to the midline and the implantation of FGF8-soaked beads rescued the migration defect toward the implantation site (Regan et al., 2009). Neugebauer and Yost (2014) reported that FGF signaling plays a role in breaking the symmetry of the brain controlling the expression of two transcription factors called *six3b* and *six7*. These genes are involved in the transcriptional repression of *lefty1*, one of the nodal left-sided targets. In detail, the knockdown of both *six3b* and *six7* leads to a bilateral expression of *lefty1* in the zebrafish dorsal epithalamus, while the overexpression of these genes represses *lefty1* in both sides of the embryo (Inbal et al., 2007). Other important evidence reported from these authors showed that FGF exerts a role also in the generation of brain asymmetry interacting with Nodal pathways (Neugebauer and Yost, 2014). Finally, the blocking of FGF signaling disrupts midline organization (Neugebauer and Yost, 2014). Overall, these data show a clear contribution of FGF to the establishment of epithalamic asymmetry, but the Nodal pathway and not FGF signaling is essential for the direction of asymmetry (Güntürkün and Ocklenburg, 2017).

Although FGF signaling is responsible to guide Nodal pathway in breaking symmetry of epithalamic structures of zebrafish brain, a critical role of the successful Nodal–mediated left-right asymmetry induction is played by Notch pathway. Notch pathway is involved in the control of cilia length and, in particular, of the cilia of an epitelium that originates from the dorsal forerunner cells at the end of zebrafish gastrulation and organizes a fluid-filled organ, called Kupffer’s Vesicle (Melby et al., 1996; Essner et al., 2005; Takeuchi et al., 2007; Lopes et al., 2010; Figure 1D). These cilia are responsible for breaking the initial symmetry generating a directional fluid flow from the Kuppfer’s Vesicle to the left side of the zebrafish and medaka embryo and to position Nodal signaling molecules on the left side. Furthermore, this flow positions *Charon*, an antagonist of *nodal* belonging to the *cerberus-like* family and under the transcriptional control of Notch signaling, on the right side (Hashimoto et al., 2004; Gourronc et al., 2007; Hojo et al., 2007). These data were also supported by earlier studies conducted by Raya et al. (2003) that showed that the bilateral injection of Notch mRNA caused the bilateral expression of *ndr2* and *pitx2*, normally expressed only on the left side, reporting for the first time a fundamental relation between Notch and Nodal signaling in the generation of asymmetry in zebrafish embryos. The involvement of Kuppfer’s Vesicle in the positioning of Nodal-relates leftward markers were also confirmed by experiment with *mother-of-snow-white* (*msw*) fish, a maternal-effect gene that control Kuppfer’s Vesicle morphogenesis and that is able to influence brain asymmetry and lateralized behaviors (Domenichini et al., 2011).

The last pathway involved in the establishment of the brain asymmetry in zebrafish is the Wnt pathway. During development, the Wnt/beta-catenin cascade acts in the lateral mesodermal plate before the induction of Nodal pathway components. The major role of this pathway in the establishment of left-right asymmetry of the brain is related to three different developmental stages of zebrafish: late gastrulation, somitogenesis and during epithalamic development (Carl et al., 2007; Hüsken and Carl, 2013; Figure 1E). Carl et al. (2007) reported that mutations of *axin/masterblind*, a wnt inhibitor expressed at the end of gastrulation in the forming anterior neural plate, or the transient wnt inhibition with lithium chloride leads to zebrafish embryos that showed a loss of the asymmetrical distribution of Nodal-related genes in the brain, but not in the lateral mesoderm, suggesting a role of Wnt pathway in the establishment of left-right asymmetry in the brain. The mechanism through which Wnt pathway influences Nodal signaling is still partially unclear but the hypothesis converges on *six3* gene that is downstream Wnt signaling at the end of gastrulation in the anterior neural plate and works as repressor of Nodal left-sided target genes in the forming neural tube (Carl et al., 2002; Lagutin et al., 2003; Inbal et al., 2007; Sagasti, 2007; Hüsken and Carl, 2013). During somitogenesis, a second involvement of Wnt signaling contributes to the development of Kupffer’s Vesicle mediating the activation of the ciliogenic transcription factor *foxj1a* and contributing to the positioning of Nodal related genes on the left side of the embryos reinforcing the action played by Notch signaling (Caron et al., 2012; Hüsken and Carl, 2013). Finally, during epithalamic and habenular development, Wnt signaling mediates the activation of the transcription factor *tcf7l2* that is expressed in the dorsal habenular nuclei (left and right) and mediates the ability of dorsal habenular neurons to respond appropriately to signals deriving from the environment into they are born in a left-right manner (Hüsken et al., 2014).

### Zebrafish as Tool to Study Brain Asymmetry

Although zebrafish has contributed to establish and clarify several developmental processes that generate asymmetric structures in vertebrate brain, this species has also made it possible to adopt different strategies to study and control the generation of such asymmetries. Over the years, in fact, researchers have developed different experimental protocols in order to establish brain asymmetry exploiting chemical, environmental (non-genetic), surgical and genetic factors.

We have already previously mentioned chemical compounds able to influence the generation of asymmetrical brain structures in fish, some of which are antagonist of the major molecular pathway that contribute to the embryonic development of vertebrates. SB431542, a specific inhibitor of TGF-beta type I receptors, is able to downregulate the expression of left-sided Nodal-related factors (*pitx2* and *lefty1)* generating in the dorsal epithalamus symmetric habenular nuclei instead of asymmetric structures (Roussigné et al., 2009). SU5402, a drug that inhibits FGF receptors, acts blocking the migration of parapineal cells from the midline to the left side of the epithalamus generating a symmetric distribution of *lefty1* and symmetric habenulae (Regan et al., 2009). IWR-1 is a stabilizer of *axin* that mediates the degradation of beta-catenin (*wnt* effector) generating a double-left habenular phenotype in the zebrafish larvae (Dreosti et al., 2014).

Modulation of brain and behavioral asymmetry in zebrafish embryos can be also induced by change in the environment: light stimulation and temperature. As described for other vertebrates, also in zebrafish asymmetry is modulated by light (Andrew et al., 2009b; Budaev and Andrew, 2009). Zebrafish embryos grown in the dark during the first day of development showed a reversed lateralized behavioral pattern, suggesting a contribution of the light in the development of brain asymmetry with, possibly, an involvement of habenular asymmetry in this process (Andrew et al., 2009b; Budaev and Andrew, 2009). Another environmental factor that can influence zebrafish brain laterality is temperature. Data reported a randomization of habenular nuclei direction followed by a loss of lateralization in the ability to respond to sensory stimuli (visual and olfactory) in zebrafish embryos that were grown at 22°C for 3–4 h at the tailbud stage instead of 28°C (Roussigné et al., 2009; Dreosti et al., 2014).

Experiments of surgical ablation of parapineal using two-photon laser microscopy in reporter zebrafish lines (e.g., FoxD3:GFP lines) were optimized to study the involvement of parapineal cells in the establishment of left-right asymmetry of the zebrafish brain. This surgical procedure gives rise to zebrafish embryos with a symmetric double-right phenotype that contributes to establish the influence of epithalamic cells in the asymmetric distribution of Nodal-related left-sided genes and the generation of asymmetrical habenular nuclei (Concha et al., 2003; Aizawa et al., 2005; Gamse et al., 2005; Bianco et al., 2008) and to clarify the involvement of habenular nuclei in response to visual (left) or olfactory (right) stimuli (Dreosti et al., 2014).

The transparency of embryos and larvae, and the possibility of an easy manipulation and accessibility to transgenesis of the embryos represent the most important advantages of using zebrafish in biomedical research and neuroscience. In the last decade, new genetically encoded optical tools and fluorescent sensors have been generated to monitor neural development and neuron activity with a very high space-time resolution (Keller and Ahrens, 2015). In fact, zebrafish represents a good compromise between system complexity and practical simplicity. In order to study the development and function of left-right asymmetry in the brain, genes expressed in the subnuclei of different regions of the zebrafish brain were identified and used as markers and transgenes. For example, Gamse et al. (2003, 2005) defined six molecular distinct domains in the zebrafish larval habenula using a combinatorial expression of *potassium channel tetramerization domain containing genes* (*kctd12.1*, *kctd12.2*, and *kctd8*). A combined approach that implies the use of a transgenic line [Tg(*brn3a–hsp70:GFP*)] and an expression marker (*kctd12.1*) helped to clarify the boundaries between the medial (*brn3a*) and lateral (*kctd12.1*) habenula (Aizawa et al., 2005), the neurotransmitters map of the asymmetric dorsal habenular nuclei (deCarvalho et al., 2014), and the extension of axons and asymmetric connections of the habenular compartments toward zebrafish telencephalic hemispheres and ventral midbrain (Beretta et al., 2017). Another important tool to study brain asymmetry and laterality was represented by Tg(foxD3:GFP) fishes that express the *green fluorescent protein (GFP)* under the control of the *foxd3* promoter, a marker of pineal and parapineal precursors and neurons. This transgenic line has been widely used to study epithalamic asymmetry (Bianco et al., 2008; Garric et al., 2014; Hüsken et al., 2014; Khuansuwan et al., 2016), involved signaling pathways (Concha et al., 2000, 2003; Gamse et al., 2003; Carl et al., 2007; Roussigné et al., 2009; Regan et al., 2009; Clanton et al., 2013), epithalamic asymmetric connections (Aizawa et al., 2005; Gamse et al., 2005; Bianco et al., 2008; Miyasaka et al., 2009; Krishnan et al., 2014; Turner et al., 2016), and the relationships between brain and behavioral asymmetries (Agetsuma et al., 2010; Dreosti et al., 2014; Krishnan et al., 2014; Facchin et al., 2015). Finally, Lekk et al. (2019) has recently generated a CRISPR/Cas9 transgenic line in which the knock-out of *sox1a* give rise to the first genetic right isomerism of the habenula (Lekk et al., 2019).

### Lesson From Other Fish and Evidence of Telencephalic Lateralization (Large Scale Fish)

Brain asymmetry has also been reported in different species of fishes. In 2009, Reddon and colleagues showed continuous variation of habenular asymmetry that correlated with growth rate in the cichlid fish *Geophagus brasiliensis*, with leftward bias in low growing fishes and larger right habenula in the faster growing individuals, also finding a positive correlation between the habenular structures and behavioral asymmetries. Similar results were obtained in another cichlid fish, *Amatitlania nigrofasciata* (Gutiérrez-Ibáñez et al., 2011). Moreover, genetic variations affecting brain asymmetry were also reported in the adult Chinook salmon (*Onchorhynchus tshawytscha*) (Wiper et al., 2017). But the most well-documented case of a relationship between behavioral lateralization and morphological asymmetry in fish is represented by *Peridossus microlepis*, a cichlid fish that is endemic of Lake Tanganyika in Africa (Hori, 1993). This species is characterized by individuals that attack their prey on the flank with a side preference associated with a morphological asymmetry of the mouth that seems to be genetically encoded (Lee et al., 2015; Raffini et al., 2017). Lee et al. (2015) used a genome-wide RNA sequencing approach and showed that different regions of the brain (such as optic tectum, telencephalon, hypothalamus, and cerebellum) displayed a different molecular signature and that some of the genes expressed in the paired brain regions (e.g., telencephalon and optic tectum) were differentially expressed between the two hemispheres suggesting that specific asymmetries in genes expression could be associated with asymmetric behavior.

Taken together, these data open the possibility to include innovative powerful tools, such as genome-wide RNA sequencing approaches, to further investigate the correlation between brain and behavioral asymmetries in fish to in order to link ecological traits to genetics and extend the results to other vertebrates.

### The Zebrafish as a Model to Investigate the Relationship Between Structural and Functional Brain Asymmetries

There is considerable evidence that zebrafish exhibit several lateralized behaviors. Adults observed in a detour task showed a left-eye bias to view an empty space or familiar environment, but they used the right eye to view a novel complex environment. Similarly, zebrafish were found to prefer to use the left eye to view a familiar social species and the right eye to view a not familiar and potentially competitive species such as the fighting fish (Miklosi et al., 1997), suggesting that the right eye is preferentially used to look at stimuli that elicit strong reactions. Adults also exhibited a preference for using the right eye when they had to approach a target to bite (Miklosi and Andrew, 1999). When larval zebrafish entered a novel lit environment after gradually dimming the light in their own compartment, they showed a strong tendency to turn to the left (Watkins et al., 2004). However, when the light was rapidly turned off, they preferentially turned to the right, showing a locomotor behavior similar to a startle-response (Burgess and Granato, 2007). Zebrafish larvae also favored the left eye for viewing their own reflection although differences in behavior have been observed in different strains (Sovrano and Andrew, 2006). Moreover, larvae had an initial preference to use the left eye to look at a novel object and then they shifted to the right eye, presumably when the object became familiar. The right-eye bias was maintained even when the fish were presented with the same object after 2 h, thus providing evidence of long-term memory (Andersson et al., 2015). Interestingly, in larval zebrafish, the general preference for the use of the left eye during inspection of its own mirror image is punctuated by a series of very short duration events and with precise cyclicality (about 160 s), during which the right eye (left hemisphere) is used instead (Andrew et al., 2009a). Similar phenomena have been observed in the processes of consolidation in memory in higher vertebrates, which are hypothesized to be related to processes that take place in the nervous system of “recording” of memory traces located in the right and left hemispheres (Andrew, 2002a). Andrew et al. (2009a) has also documented the existence of anomalies in the duration and periodicity of the events of use of the right eye also in mutant zebrafish strains characterized by inversion of parapineal asymmetries.

In the last two decades much effort has been devoted to investigate the relationship between brain and behavioral asymmetries. One advantage of using zebrafish in this research field is that it offers experimental manipulations that cannot be used in humans for ethical and practical reasons. Different strategies have been adopted to modify the L-R epithalamic asymmetry. For instance, Barth et al. (2005) used larvae from a genetic line known as *frequent-situs-inversus* (*fsi*) in which the parapineal was located to the right side of the pineal organ in about 25% of individuals (rather than 3% as reported in wild-type) and this neuroanatomical symmetry was concordant with visceral reversal of gut and heart. Fish with left (Lpp) and right parapineal (Rpp) observed in different assays showed reversed behavioral asymmetries in the mirror test and in the biting test. In the first test, Lpp larvae used the left eye to view their mirror image, whereas Rpp larvae used the right eye. In the biting test, Lpp adults looked at the stimulus to bite with the right eye and the Rpp used more often the left eye. Despite, there was no difference in turning behavior when larvae entered a novel environment between the two groups, their latency to emerge differed and it was higher in Lpp than in Rpp. Taken together, these results suggest that there might be a causal relationship between epithalamic asymmetries, some lateralized behaviors and behaviors related to fear/anxiety. Change in the frequency of reversed asymmetry in the epithalamus can also been obtained as a result of artificial selection for the eye used in the mirror test. Zebrafish selected for five generations for right-eye use showed a significant increase of reversed asymmetry whereas selection for left-eye led to a decrease of asymmetry (Facchin et al., 2009a). In a subsequent study, larvae from the line selected for the right-eye use were sorted for the left or right position of the parapineal using the foxD3:GFP marker and were then observed when adults in a series of laterality tests (i.e., eye used in predator inspection, rotational preference, and turning direction in the dark) (Dadda et al., 2010a). Opposite lateralized behaviors were observed between the Lpp and Rpp. Furthermore, differences in some personality traits were found as fish with Rpp were bolder in certain contexts, as reported by Barth et al. (2005). Along similar lines, Domenichini et al. (2011) found that Lpp and Rpp adults showed a reversed pattern for the eye used in the detour task to scrutinize a predator and their own mirror image but no difference was observed when they were presented with a neutral stimulus (i.e., a plant).

However, Facchin et al. (2009b) provided contrasting results when compared larvae with reversal of epithalamic asymmetry, induced by injection of *southpaw* antisense morpholino, with control larvae with typical L-R pattern. No difference was found in the mirror test and in the C-start response following an acoustic/vibrational stimulus or after presentation of a lateralized stimulus. Despite behavioral responses were similar, larvae with right parapineal showed a significant delay in the onset of navigation and reduced swimming speed. Consistent with these findings in larvae, adults with reversed L-R brain asymmetry were discovered to manifest different behaviors indicative of anxiety: Rpp spent more time in the bottom section of a novel tank, showed reduced explorative behavior in the mirror test, increased latency in exiting from a confined box and higher cortisol levels compared to Lpp (Facchin et al., 2015). The scenario that emerges from these studies is far from simple as the disruption of directionality in the zebrafish epithalamus clearly seems to affect some, but not all lateralized behaviors and also plays a role in regulating stress response. It is worth noting that discrepancies among studies may be ascribed to different strains used, different methods adopted and different ways of analyzing data making it clear the need of standardized protocols to enhance reproducibility.

Finally, manipulations of brain asymmetry also affect sensory responses to light and odor. Imaging of the neural activity of dorsal habenula neurons (dHb) showed that light mainly activated the neurons in the left dHb, whereas odor mainly activated the neurons in the right dHb. This pattern of sensory processing was found to be opposite in fish with reversed L-R asymmetry. Furthermore, loss of asymmetry in fish with double -left- or double-right-sided brains caused loss of response to both stimuli suggesting that the alteration of brain lateralization could be causative of cognitive disfunctions rather than their consequences (Dreosti et al., 2014).

## Conclusion

It is clear that brain lateralization is a widespread and well-conserved phenomenon in vertebrates (see Vallortigara and Rogers, 2020). Research on fish has proved to be valuable in understanding its biological function and the evolutionary significance. Whether brain lateralization is a homologous trait inherited by a common ancestor in vertebrates or if it has emerged more than once as result of convergent evolution has not yet been determined.

Boorman and Shimeld (2002) suggested that structural asymmetry has probably evolved numerous times in animals, but its frequent occurrence may reflect conserved molecular mechanisms. Since all members of Bilateria (i.e., animals with bilateral symmetry) share directional asymmetries, it is plausible to hypothesize, by parsimony, that these traits are homologous. If we focus our attention on the vertebrates, research on fish can help to answer this question. Fish are the most ancient vertebrates (first fossils date back to ∼500 million years ago, Shu et al., 1999), represent half of the vertebrate species on the planet (Diana, 2003) and have adapted to live in almost every aquatic niche. Consequently, they represent a useful tool to investigate the role of phylogeny and ecology in the development of brain lateralization given the complexity of their social and physical environment and the diversity of the existing species. The ubiquity of morphological asymmetry associated to functional asymmetry in fishes may indicate a monophyletic origin and may have been present in the ancestors of vertebrates. Furthermore, evidence of asymmetry in coelacanths and lungfish which share a common ancestor with terrestrial tetrapods (i.e., amphibians, reptiles, birds, and mammals), support the idea that they inherited this trait from fish (Hori et al., 2017).

In humans, an increasing number of studies has noticed an association between atypical pattern of cerebral asymmetry and cancer (Sandson et al., 1992; Klar, 2011), immune reactivity (Neveu, 2002), autism (Escalante-Mead et al., 2003; Herbert et al., 2005), schizophrenia (Klar, 1999; Ribolsi et al., 2009), and dyslexia (Heim and Keil, 2004; Wijers et al., 2005).

Despite rapid and continuous progress has been made in neuroimaging, neurostimulation and genetic techniques used to investigate lateralization in humans, it remains difficult, for ethical and practical reasons, to assess the role of the environmental stimulation and of the extent of genes contribution to the development of brain asymmetry. Among animal models, the zebrafish has rapidly become a powerful species to investigate lateralization at different level of complexity, from genes, to structural and functional asymmetry, providing insights into the establishment of brain lateralization and the molecular processes involved. The combination of behavioral analysis, imaging and cutting-edge molecular genetic techniques represents a unique approach to investigate gene-by-environment interaction effects, how genetically encoded asymmetry may chance across the lifespan and how anatomical asymmetries are linked to behavior. Research on fish and, in particular on zebrafish, is of paramount importance to increase our comprehension of the biological relevance of brain lateralization and to understand how defects in brain asymmetry contribute to neurological disorders and pathologies in humans and other animals.

## Author Contributions

MM, AM, GV, and VS conceived and designed organization of the manuscript. All authors contributed to the manuscript writing.

## Conflict of Interest

The authors declare that the research was conducted in the absence of any commercial or financial relationships that could be construed as a potential conflict of interest.
